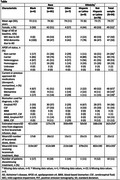# Real‐World Use of Lecanemab With Consideration of Race, Ethnicity, and Geographical Diversity

**DOI:** 10.1002/alz70861_108602

**Published:** 2025-12-23

**Authors:** Jose Soria‐Lopez, Michael Henry Rosenbloom, Marwan N. Sabbagh, Cara Leahy, Gregory Cooper, Samuel Giles, Martin Sadowski, Curtis Schreiber, Paul E Schulz, David C Weisman, Christian J Camargo, Brooke Allen, Katie Jamison, Daryl Jones

**Affiliations:** ^1^ The Neuron Clinic, San Diego, CA USA; ^2^ University of Washington Memory and Brain Wellness Center, Seattle, WA USA; ^3^ University of Washington Alzheimer's Disease Research Center, Seattle, WA USA; ^4^ Barrow Neurological Institute, Phoenix, AZ USA; ^5^ Memorial Healthcare Institute for Neuroscience, Owosso, MI USA; ^6^ Norton Neuroscience Institute, Louisville, KY USA; ^7^ Memory Treatment Centers, Jacksonville Beach, FL USA; ^8^ New York University Langone Health, New York, NY USA; ^9^ Missouri Memory Center, Citizens Memorial Hospital, Bolivar, MO USA; ^10^ John P. and Kathrine G. McGovern Medical School at UTHealth, Houston, TX USA; ^11^ Abington Neurologic Associates, Abington, PA USA; ^12^ University of Miami Miller School of Medicine, Miami, FL USA; ^13^ Roaring Fork Neurology, Basalt, CO USA; ^14^ Eisai Inc, Nutley, NJ USA; ^15^ Eisai Inc., Nutley, NJ USA

## Abstract

**Background:**

Lecanemab‐irmb (LEQEMBI®) is indicated for the treatment of patients with Alzheimer’s disease (AD) in the mild cognitive impairment or mild dementia stage. Racial and ethnic disparities in access, care, and outcomes exist in AD. This study aimed to describe real‐world experience with lecanemab in racially, ethnically, and geographically diverse patients with early AD in the United States.

**Method:**

This multicenter, retrospective case series and patient pathway study was conducted in 15 geographically diverse neurology clinics, each abstracting deidentified medical chart data for up to 25 patients receiving lecanemab (≥7 infusions) and 1 neurologist per site completing an electronic survey plus an interview. Data collected included sociodemographic characteristics, clinical characteristics, AD diagnosis, and lecanemab use, in addition to practice characteristics and norms to assess best practices. This interim analysis (cutoff date: April 11, 2025) includes ∼25% of the expected total study population (final data cut: May 23, 2025). The protocol received central institutional review board exemption.

**Result:**

Of 94 CRFs completed, 6 cases were Black, 81 White, and 8 indicated as Other; clinical characteristics are summarized in the Table. Baseline characteristics were mostly balanced between the racial and ethnic groups, except all Black patients were in the mild AD dementia stage and a higher proportion were *APOE ε4* carriers, and the estimated mean time from diagnosis to first lecanemab infusion was higher. In terms of geographical diversity, across the 94 patient cases, a total of 43 (46%) patients were from an urban area, 32 (34%) from a rural area, and 14 (15%) from a suburban area.

**Conclusion:**

Black individuals initiated lecanemab at a later stage of disease (mild AD dementia), which may indicate a delay in diagnosis and/or treatment initiation (interim data analysis). Owing to the small sample size across different groups at this interim analysis, interpretation is limited. The full data set analysis (data cutoff: May 23, 2025) will further evaluate differences across these patient groups and explore patient pathways and initiatives for underserved populations.